# “Fei Yan No. 1” as a Combined Treatment for COVID-19: An Efficacy and Potential Mechanistic Study

**DOI:** 10.3389/fphar.2020.581277

**Published:** 2020-10-08

**Authors:** Zhongzhu Ai, Shanshan Zhou, Weinan Li, Mengfan Wang, Linqun Wang, Gangming Hu, Ran Tao, Xiaoqin Wang, Yinfeng Shen, Lihan Xie, Yuanming Ba, Hezhen Wu, YanFang Yang

**Affiliations:** ^1^Faculty of Pharmacy, Hubei University of Chinese Medicine, Wuhan, China; ^2^First Clinical College, Hubei University of Chinese Medicine, Wuhan, China; ^3^Nephrology Department, Hubei Provincial Hospital of TCM, Hanchuan, China; ^4^Nephrology Department, Hubei Provincial Traditional Chinese Medicine Research Institute, Wuhan, China; ^5^TCM Department, Renmin Hospital of Hanchuan, Hanchuan, China; ^6^Surgical Department, Hubei Provincial Hospital of TCM, Wuhan, China; ^7^Surgical Department, Hubei Provincial Traditional Chinese Medicine Research Institute, Wuhan, China; ^8^Nephrology Department, The Central Hospital of Wuhan, Wuhan, China

**Keywords:** Fei Yan NO1, COVID-19, SARS-CoV-2, clinical efficacy, network pharmacology

## Abstract

There has been a large global outbreak of coronavirus disease 2019 (COVID-19), caused by severe acute respiratory syndrome coronavirus 2 (SARS-CoV-2), representing a major public health issue. In China, combination therapy, including traditional Chinese medicine (TCM) as a treatment for COVID-19 has been used widely. “Fei Yan No. 1” (QFDYG) is a formula recommended by the Hubei Government to treat COVID-19. A retrospective study of 84 COVID-19 patients from Hubei Provincial Hospital of TCM and Renmin Hospital of Hanchuan was conducted to explore the clinical efficacy of QFDYG combination therapy. TCMSP and YaTCM databases were used to determine the components of all Chinese herbs in QFDYG. Oral bioavailability (OB) ≥ 30% and drug-like (DL) quality ≥ 0.18 were selected as criteria for screening the active compounds identified within the TCMSP database. The targets of active components in QFDYG were determined using the Swiss TargetPrediction (SIB) and Targetnet databases. The STRING database and the Network Analyzer plugin in Cytoscape were used to obtain protein-protein interaction (PPI) network topology parameters and to identify hub targets. Gene Ontology (GO) enrichment was conducted using FunRich version 3.1.3, and Kyoto Encyclopedia of Genes and Genomes (KEGG) pathway enrichment using ClueGO version 2.5.6 software. PPI and compound-pathway (C-T) networks were constructed using Cytoscape 3.6.0. Compared with the control group, combined treatment with QFDYG resulted in a significantly higher rate of patients recovering from symptoms and shorter the time. After 14 days of treatment, QFDYG combined treatment increased the proportion of patients testing negative for SARS-CoV-2 nucleic acid by RT-PCR. Compared with the control group, promoting focal absorption and inflammation as viewed on CT images. GO and KEGG pathway enrichment indicated that QFDYG principally regulated biological processes, such as inflammation, an immune response, and apoptosis. The present study revealed that QFDYG combination therapy offered particular therapeutic advantages, indicating that the theoretical basis for the treatment of COVID-19 by QFDYG may play an antiviral and immune response regulation through multiple components, targets, and pathways, providing reference for the clinical treatment of COVID-19.

## Introduction

Since December 2019, coronavirus disease 2019 (COVID-19), caused by infection with the SARS-CoV-2 virus, has spread to most countries and regions around the world (Zhu et al., 2020). In common with two other highly pathogenic coronaviruses, SARS-CoV and MERS-CoV, SARS-CoV-2 is also a β-coronavirus (Xu et al., 2020), principally transmitted by close contact and through the respiratory tract, resulting in severe respiratory illness and even death. The main clinical manifestations of COVID-19 are fever, cough. A small number of patients also display symptoms that include sore throat, stuffy or runny nose, muscle pain, and diarrhea. A number of patients rapidly progress to acute respiratory failure or acute respiratory distress syndrome (ARDS) (Chen N. et al., 2020). Because of the scale of the outbreak, the World Health Organization (WHO) announced a public health emergency of international concern (PHEIC) on 30^th^ January 2020 (Sohrabi et al., 2020). According to the recent data from the WHO, the number of global infections has passed 27 million, with a death toll greater than 880,000, threatening 214 countries. Therefore, there is a pressing need to establish a specific and effective therapeutic schedule for the treatment of COVID-19.

Traditional Chinese medicine (TCM), which has a history of successful therapy over thousands of years, has made a substantial contribution to the health and prosperity of the entire nation. TCM has accumulated the rich experience of fighting emerging infectious diseases over a considerable period of time with a solid theoretical foundation for their treatment (Luo et al., 2020). Although COVID-19 is a novel infectious disease caused by SARS-CoV-2, TCM has been used to successfully treat similar syndromes caused by coronaviruses. In addition, TCM has played an important role in combating coronavirus pneumonia, such as that caused by SARS and MERS, in addition to H7N9 avian influenza, over the last two decades (Ren et al., 2020). In 2003, SARS broke out in China. Studies found that the earlier an intervention with TCM was initiated, the lower the rate of mortality and incidence of complications was observed (Du et al., 2020).

Following the outbreak of COVID-19, China’s National Health Commission proposed a diagnosis and treatment program through the integration of TCM with modern medicine, as no antiviral drug around the world had at that time been shown to be effective. The combination treatment of COVID-19 using traditional Chinese medicine is widely used in China. TCM marked improvement of symptoms and shortened the disease course, and also better control of fever, quicker clearance of chest infection and other symptoms. The symptoms of the majority of COVID-19 patients improving markedly, demonstrating its capacity to promote recovery (Yang et al., 2020a). Of the proposed diagnoses and treatments, a TCM formula termed “Fei Yan No. 1” (QFDYG) was recommended by the Hubei Headquarters for the Prevention and Control of Novel Coronavirus Pneumonia Epidemic and approved by the Hubei Provincial Drug Administration (Z20200003) for the treatment of COVID-19. It consists of 13 traditional Chinese medicines, including *Bupleurum chinense* DC. (Chaihu), *Scutellaria baicalensis* Georgi (Huangqin), *Pinellia ternata* (Thunb.) Breit. (Banxia), *Codonopsis pilosula* (Franch.) Nannf. (Dangshen), *Trichosanthes kirilowii* Maxim. (Gualou), *Areca catechu* L. (Binglang), *Amomum tsao-ko* Crevost et Lemaire (Caoguo), *Magnolia officinalis* Rehd. et Wils. (Houpo), *Anemarrhena asphodeloides* Bge. (Zhimu), *Paeonia lactiflora* Pall. (Chishao), *Glycyrrhiza uralensis* Fisch. (Gancao), *Citrus reticulata* Blanco (Chenpi), and *Polygonum cuspidatum* Sieb. et Zucc. (Huzhang). Production quality control standards for QFDYG have been established and it has been widely used for the clinical treatment of COVID-19 patients in Hubei Province. The process of preparation of QFDYG can be described as follows: prescribed quantities of the Chinese Traditional Medication were weighed out for decoction, extracted twice in water using an automatic digital instrument, concentrated under reduced pressure, spray-dried to obtain a powder, to which auxiliary materials were added. It was granulated using a dry process and then packaged. High performance liquid chromatography (HPLC) was used to measure its 6 principal components and thin-layer chromatography (TLC) used to detect the three components of greatest concentration in QFDYG, from which quality control standards were established. All materials and operations met the requirements of the Chinese Pharmacopoeia (*2015 edition*). Although QFDYG was used for the treatment of COVID-19, its clinic effectiveness remains uncertain, and the bioactive components and their mechanism of antiviral action remain unclear.

Network pharmacology is based upon the theories of system biology and multi-directional pharmacology (Zhou Y. et al., 2020). It allows the construction of biological networks and network visualization analysis of bioactive components, hub targets, and signaling pathways to elucidate the complex relationships between drugs and diseases (Zhou S. et al., 2020). Network pharmacology can reveal the mechanisms of action of a TCM formula with multiple components and target characteristics (Li and Zhang, 2013).

**Figure 1:**
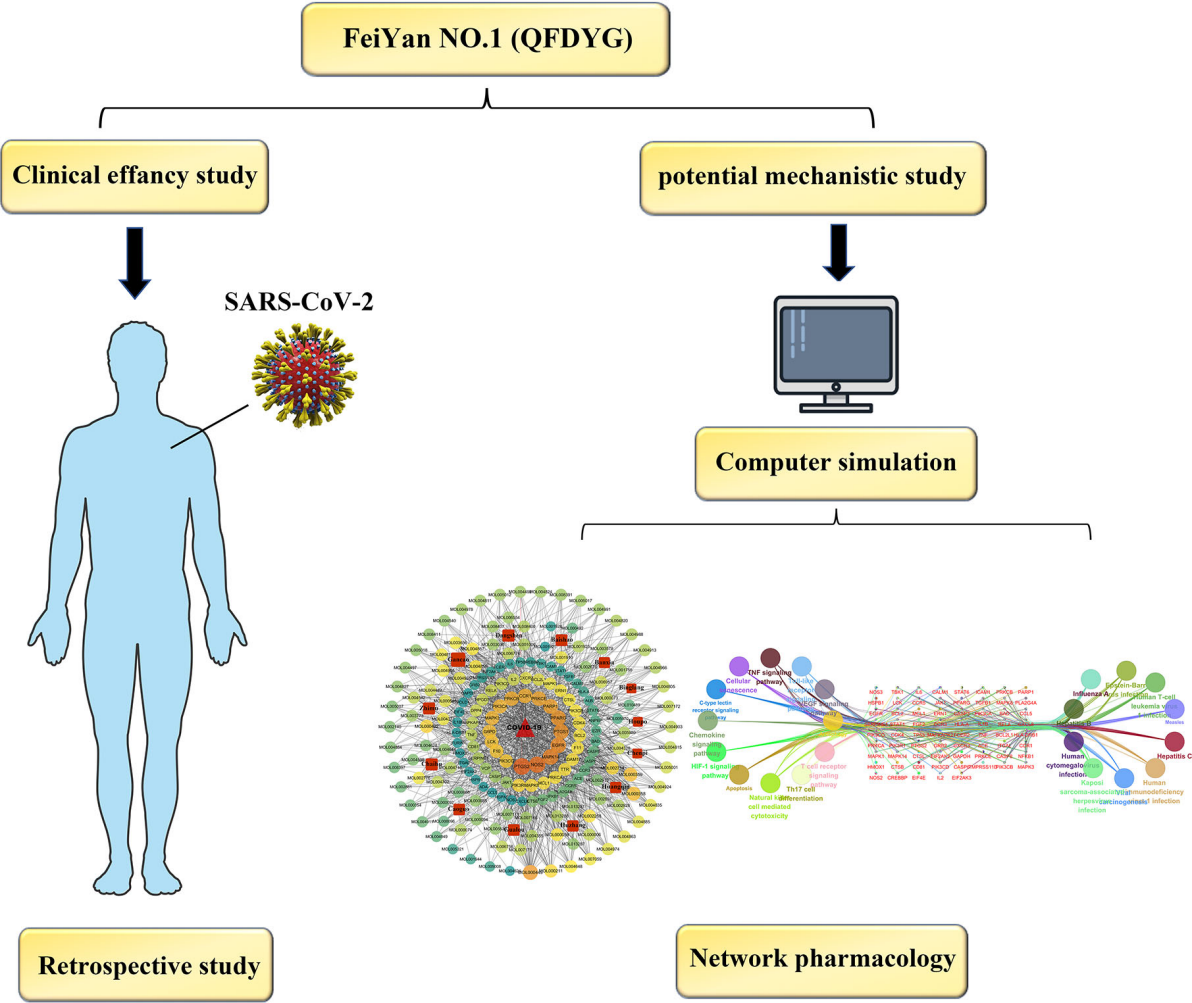
Clinical efficacy.

Therefore, as a first step, studies that used combination therapy of COVID-19 patients with QFDYG were systematically reviewed in order to explore the evidence for its clinical efficacy. Based on definite curative outcomes, network pharmacology was used to explore the potential bioactive components and therapeutic mechanisms of QFDYG in treating COVID-19. In the present study, we aimed to establish the evidence as a reference for the optimal clinical practice of treatment of COVID-19 using QFDYG and determine the therapeutic mechanisms. A graphical abstract of the study is displayed in **Figure 1**.

## Materials and Methods

### Clinical Efficacy Study

#### Study Design and Participants

The design was a retrospective study and was approved by the Institutional Ethics Board of Hubei Province Hospital of TCM (HBZY2020-C02-01), for which the requirement for written informed consent was waived. Patients included all those aged 18 years old or greater of either sex. The diagnosis of COVID-19 was confirmed by a positive nucleic acid RT-PCR test for SARS-CoV-2. If an individual’s sample were tested positive, it will be included in further study. In general, adults with COVID-19 can be grouped into the following categories of illness severity **(****Table 1****)**, in accordance with the “*Protocol for Diagnosis and Treatment of COVID-19 guidelines (4^th^ edition)*,” issued by the National Health Commission of China (General Office Of The National Health And Health Commission, 2020).

**Table 1:** The clinical classification of COVID-19.

Clinical classification	Protocol for Diagnosis
Mild	Individuals who test positive for SARS-CoV-2 with RT-PCR. The clinical symptoms were mild. With or without fever or respiratory diseases. With or without showed obvious lung infiltrates on chest computed tomography (CT).
Severe	shortness of breath and respiratory rate (RR) ≥30 times/min. A saturation of oxygen (SpO2) ≤93% at rest. Ratio of arterial partial pressure of oxygen to fraction of inspired oxygen (PaO2/FiO2) <300 mmHg (1mmHg =0.133kPa) on room air at sea level. *In high-altitude areas (at an altitude of over 1,000 meters above the sea level), PaO_2/_FiO_2_ shall be corrected by the following formula: PaO_2/_FiO_2_ x [Atmospheric pressure (mmHg)/760]. Cases with chest imaging that showed obvious lung infiltrates >50% within 48 h. Individuals who have respiratory failure, septic shock, and/or multiple organ failure.

Key exclusion criteria included: (1) Severe systemic disease; (2) Women that were pregnant or lactating; (3) Known allergies to the investigational medication; (4) Respiratory disease caused by bacterial infection; (5) Transfer to other hospitals due to policy reasons; (6) Self withdrawal during the course of treatment; (7) Incomplete clinical data records.

Patients with confirmed COVID-19 admitted to the Hubei Province Hospital of TCM and Renmin Hospital of Hanchuan between January 27 and March 12, 2020, were included in the study.

#### Clinical Treatments and Data Collection

**Figure 2:**
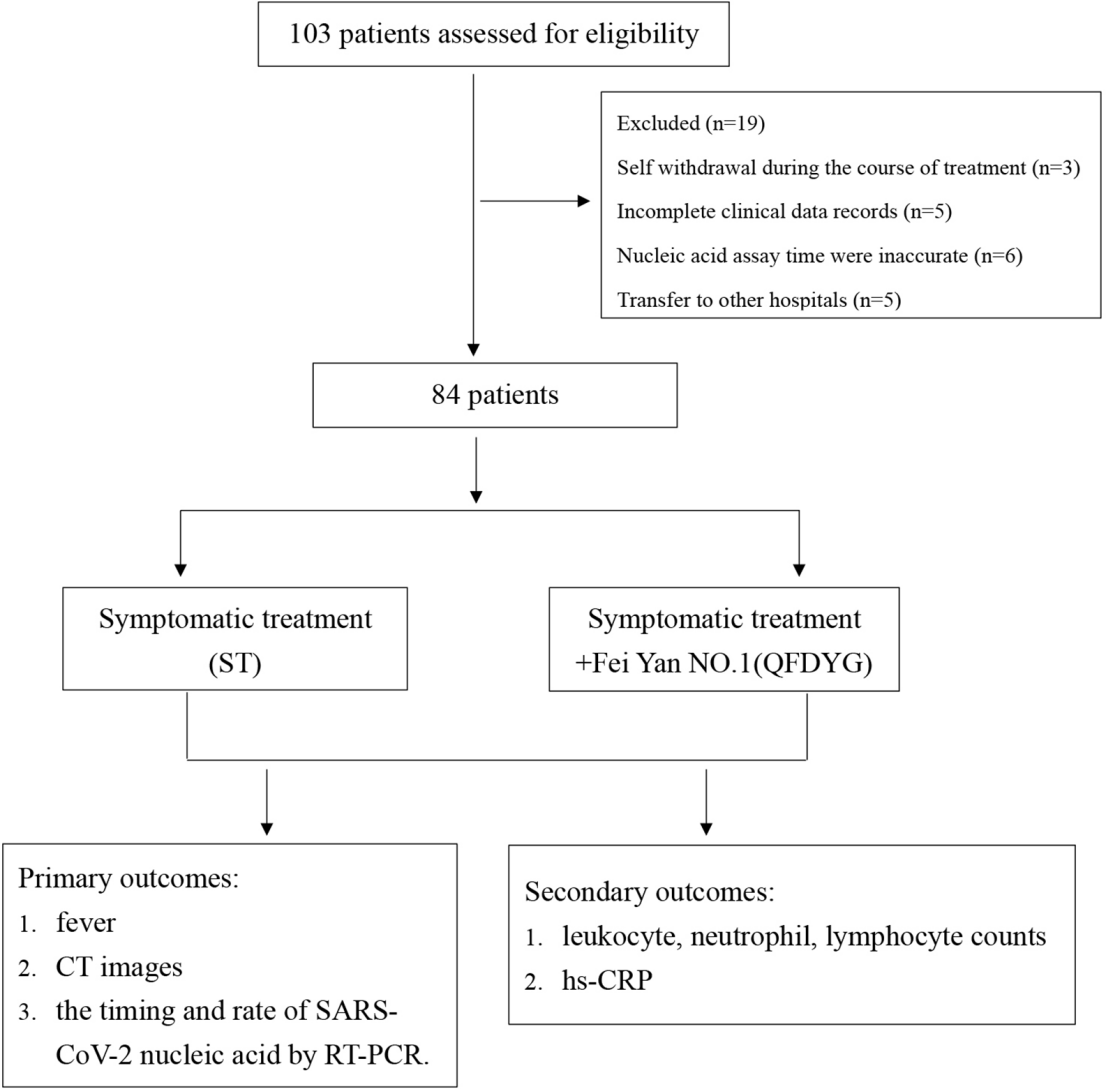
Data collection for the treatment of COVID-19 patients.

Patients were divided into two groups depending on whether they were prescribed QFDYG as a clinical treatment. In accordance with the “*Protocol for Diagnosis and Treatment of COVID-19 guidelines (4^th^ edition, 5^th^ edition and 6^th^ edition)*” (General Office Of The National Health And Health Commission, 2020) **(****Figure 2****)**, symptomatic treatment (ST) included the antivirals oral alpha interferon inhalation (50 μg twice daily), oseltamivir (500 mg twice daily), arbidol (200mg three times daily),and other treatments or interventions based on disease progression. There are no obvious differences with respect to the use of other drugs and treatments between the two groups. For ST+QFDYG group, each patient was given the same treatments as in the basic therapy plus QFDYG (18-g granules, three times daily). symptom recovery was represented by complete remission of both symptoms. Clinical cure was defined as having met all of the following criteria: recovery of body temperature for more than 3 days, symptom recovery, marked improvement in chest CT imaging, and two consecutive negative SARS-CoV-2 RNA tests (at least 1 day apart).

The clinical symptoms, treatment, and outcomes were collected from electronic medical records. Primary outcomes consisted of the rate of recovery from symptoms, the time required and rate for SARS-CoV-2 nucleic acid testing to be negative by RT-PCR and chest CT imaging. Secondary outcomes consisted of leukocyte, neutrophil, lymphocyte counts, hs-CRP. Data prior to admission was compared with that prior to discharge of the two groups, including age, sex, clinical classification, signs and symptoms, underlying disease, laboratory biochemical test results, and nucleic acid RT-PCR becoming negative.

#### Safety Evaluation of Two Groups

Previous reports of QFDYG showed that no obvious adverse reactions were found during the treatment, and the safety of clinical application was good (Ba et al., 2020). In our study, we recorded the timing, severity, duration, measures and consequence of adverse events, and the association with QFDYG.

### Analysis of Network Pharmacology

#### Screening of Bioactive Ingredients

The compounds within QFDYG were identified in the TCMSP database (https://tcmspw.com/tcmsp.php), compensating for insufficient information within the YaTCM database (http://cadd.pharmacy.nankai.edu.cn/yatcm/home). Oral bioavailability (OB) ≥ 30% and drug-like quality (DL) ≥ 0.18 were selected as criteria for screening active compounds. OB denotes the relative quantity absorbed into the bloodstream after administration through an extravascular route. DL refers to the “drug-like” qualities of a compound, used to optimize pharmacokinetics and the properties of a drug. The OB values and DL indices of the components in QFDYG were retrieved from the TCMSP database.

#### Potential Targets Intersection of QFDYG With COVID-19

Targets of the active components in QFDYG were explored using Swiss TargetPrediction (SIB) (http://www.swisstargetprediction.chand) and Targetnet (http://targetnet.scbdd.com/). The Uniprot protein sequence resource (http://www.Uniprot.org/) was used to confirm and standardize protein names. Duplicates were deleted to obtain target genes. Genes associated with COVID-19 were identified using GeneCards (https://www.genecards.org/). Intersection targets for COVID-19 and their compounds were considered potential targets. Potential targets of the active components were then imported into the STRING (http://string-db.org/) database to identify protein-protein interactions (PPIs).

#### GO and KEGG Pathway Enrichment Analysis of Candidate Targets

Gene ontology (GO) functional enrichment analysis, including molecular function (MF), biological processes (BP) and cytological components (CC), was performed by inputting all potential targets into the Functional Enrichment Analysis Tool (FunRich version 3.1.3) and Kyoto Encyclopedia of Genes and Genomes (KEGG) pathway enrichment analysis conducted using the ClueGO plugin v2.5.6 in Cytoscape.

### Network Construction and Analysis

Cytoscape 3.6.0 software was used to construct PPI and drug-compound-target (D-C-T) networks. The Cytoscape Network Analyzer plugin was used to analyze network topology, in which nodes represent targets or compounds and the size and color of the nodes indicate the degree of the node.

### Statistical Analysis

Categorical variables are described as frequency rates and percentages, and compared using χ^2^ tests between two groups. Continuous variables are described using median values (interquartile range (IQR)) or means ± SD, and compared using an independent sample t-test between two groups. All statistical analyses were performed using SPSS 25.0 software. *P <*0.05 was considered statistically significant.

## Results

### Baseline Characteristics of the Patients in the Study

Of the 103 patients assessed for eligibility, 19 were excluded, including 3 that were asymptomatic infection who stopped taking the medication by themselves, 5 cases did not undergo CT image scanning at discharge, 6 where nucleic acid assay time were inaccurate (2 of patients did not cooperate with RT-PCR test for SARS-CoV-2 testing. 4 cases were due to insufficient nucleic acid detection capabilities at that time), and 5 that had transferred to other hospitals. Therefore, a total of 84 patients confirmed to have COVID-19 participated in the study. Of these, symptomatic treatment of 49 was supplemented with QFDYG (ST+QFDYG group) while the remaining 35 patients were administered symptomatic treatment only (ST group). From the baseline characteristics displayed in **Table 2**, both groups were comparable in terms of demographic characteristics, vital signs, symptoms, and concomitant treatment. The median age of patients in the two groups was 48 years in the ST group (range: 39 - 53 years) and 57 years in the ST + QFDYG group (range: 49.5 - 65.5 years). There was no significant difference in the distribution of sex or clinical classification (*P* > 0.05). The most common underlying diseases were diabetes, hypertension, hyperlipemia, coronary disease and chronic hepatitis B. Of the recorded symptoms, cough (91.42% *vs.* 91.83%), fever (85.71% *vs.* 83.67%) and chest CT (88.57% *vs.* 91.84%) were the most common in the two groups, respectively.

**Table 2:** Baseline characteristics and symptoms of the groups with or without QFDYG.

	ST(n = 35)	ST+QFDYG (n = 49)	*P*
Age, years	48 (39, 53)	57 (49.5, 65.5)	0.137
Sex (male/female)	22/13	28/21	0.599
Clinical classification (mild/severe)	31/4	37/12	0.133
**Signs and symptoms**			
Fever (> 37.2°C)	30 (85.71%)	41 (83.67%)	0.799
Cough	32 (91.42%)	45 (91.83%)	0.947
Mild insomnia	29 (82.86%)	43 (87.76%)	0.527
Chest CT	31(88.57%)	44 (91.84%)	0.858
**Laboratory indices**			
Leukocyte count (×10^9^/L)	4.23 (3.86, 4.79)	6.23 (4.93, 9.26)	0.058
Neutrophil count (×10^9^/L)	2.57 (2.45, 3.35)	4.69 (3.40, 7.36)	0.024
Lymphocyte count (×10^9^/L)	1.38 (0.71, 1.38)	1.19 (1.06, 1.21)	0.79
Hs-CRP (mg/L)	9.00 (9.00, 12.29)	9.70 (2.40, 9.80)	0.886
**Underlying diseases**			
Diabetes	8 (22.86%)	13 (26.53%)	0.701
Hypertension	9 (25.71%)	12 (24.49%)	0.898
Hyperlipemia	5 (14.29%)	8 (16.33%)	0.799
Coronary disease	3 (8.57%)	5 (10.20%)	0.802
Chronic hepatitis B	2 (5.71%)	4 (8.16%)	0.693

### Comparison of Treatment Responses Between the Groups With or Without QFDYG

**Figure 3:**
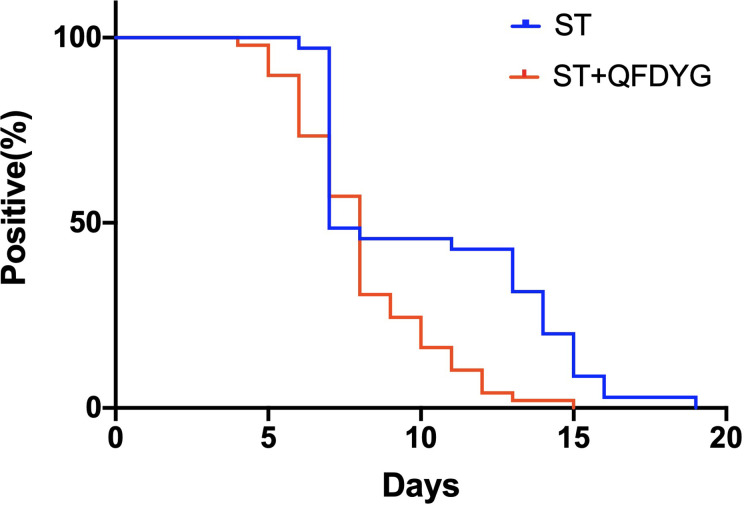
SARS-CoV-2 nucleic acid by RT-PCR between two groups (*P* < 0.05).

**Figure 4:**
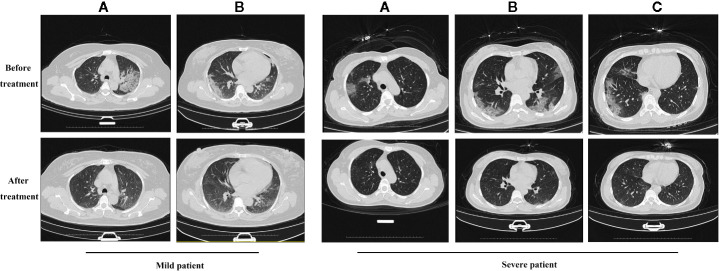
The results of chest CT scan with QFDYG combined therapy. A, B and C represent different slices. Mild patient: A 55-year-old female with bilateral pulmonary infiltrate prior to treatment with ST+QFDYG observed in A and B slices. Clear absorption of bilateral pulmonary infiltrate was observed by day 9. Severe patient: A 50-year-old female with shortness of breath and hypertension at the initiation of treatment with ST+QFDYG. Images display bilateral pulmonary infiltrate in A, B and C slices over an area larger than that of a mild patient. Marked absorption of the bilateral pulmonary infiltrate was observed by day 11, with only a few small patches of faint shadow remaining.

In the primary outcomes, the rate of symptom recovery was greater in the ST + QFDYG group compared with the ST group. Furthermore, the mean duration of recovery from fever (7.6 days *vs.*3.8 days, *P* < 0.05) was significantly shorter in the ST + QFDYG group. A nasopharyngeal swab test for SARS-CoV-2 nucleic acid by RT-PCR is considered the most important indicator of a treatment effect. The time for a negative RT-PCR result to be recorded was shorter in the ST+QFDYG group than in the ST group (7.0 days *vs.* 14.0 days, *P* < 0.05). After 14 days, coronavirus was not detected in 47 out of 49 (95.92%) and 28 out of 35 (80.00%) patients in the ST+QFDYG and ST groups, respectively (*P* < 0.05) (**Figure 3**). Chest CTs indicated that the majority of patients had apparent lung infiltrates prior to admission (88.57% *vs.* 91.84%), with ground glass or flocculent shadows observed in the lungs of patients with both mild and severe disease. Manifestations in the chest as observed by computed tomography in mild and severe patients are displayed in **Figure 4**.

In 84 cases, the leukocyte count of 18 patients and the neutrophil count of 16 patients were not in the normal range and returned to normal after treatment. The lymphocyte count of 33 patients were lower than the normal value. After treatment, the lymphocytes increased, which was significantly different (*P* < 0.05) (**Table 3**). Median hs-CRP (9.00 *vs.* 9.70) was significantly higher than the normal reference range prior to hospitalization, which decreased significantly (*P* < 0.05), close to normal, following ST+QFDYG treatment.

**Table 3:** Comparison of treatment responses between the groups with or without QFDYG.

	Reference range	ST (n=35)	ST+QFDYG (n=49)	*P*
Leukocyte count (×10^9^/L)	3.5-9.5	5.20 (4.10, 5.20)	3.97 (3.97, 7.65)	0.01
Neutrophil count (×10^9^/L)	1.8-6.3	3.48 (3.37, 3.48)	2.60 (2.20, 3.66)	0.436
Lymphocyte count (×10^9^/L)	1.1-3.2	1.24 (0.49, 1.24)^*^	1.20 (0.96, 3.01)^*^	0.510
Hs-CRP (mg/L)	0-3	4.50 (4.50, 9.64)^*^	2.40 (1.20, 2.50)^*^	0.002
Nucleic acid RT-PCR negative, days	–	14.0 (7.0, 16.0)	7.0 (5.0, 8.0)	0.04
Fever, days	–	7.6 ± 1.7	3.8 ± 1.3	0.001

Compared with before admission data in **Table 2** *P < 0.05

### Safety of Two Groups

No significant difference in the incidence of adverse events was observed between the two groups. No serious adverse events were reported and there were few reports of abnormal laboratory blood tests (**Table 4**).

**Table 4:** Comparison of the adverse events between the groups with or without QFDYG.

Adverse events	ST (n = 35)	ST+QFDYG (n = 49)	*P*
Total	19 (54.29%)	23 (46.94%)	0.507
Nausea	8 (22.86%)	11(22.45)	0.965
Abnormal liver function	5 (14.29%)	4(8.16%)	0.371
Vomiting	1 (2.86%)	1 (2.04)	0.809
Headache	3 (8.57%)	4 (8.16)	0.947
Abnormal renal function	2 (5.71%)	3 (6.12%)	0.938

### Active Components in QFDYG

The TCMSP database was used to search for active components in QFDYG. A total of 122 active ingredients fulfilling the specified criteria, OB ≥ 30% and DL index ≥ 0.18, were identified (**Table 5**).

**Table 5:** Information of the potential components with higher OB and DL values in QFDYG.

NO.	Mol ID	Molecule Name	MW	OB (%)	DL	Classification
1	MOL001918	paeoniflorgenone	318.35	87.59	0.37	Baishao
2	MOL000449	Stigmasterol	412.77	43.83	0.76	Banxia
3	MOL002776	Baicalin	446.39	40.12	0.75	Banxia
4	MOL000004	Procyanidin B1	578.56	67.87	0.66	Binglang
5	MOL000098	Quercetin	302.25	46.43	0.28	Caoguo
6	MOL000422	Kaempferol	286.25	41.88	0.28	Chaihu
7	MOL004644	Sainfuran	286.3	79.91	0.23	Chaihu
8	MOL005815	Citromitin	404.45	86.9	0.51	Chenpi
9	MOL000006	Luteolin	286.25	36.13	0.25	Dangshen
10	MOL006554	Taraxerol	426.80	38.40	0.77	Dangshen
11	MOL002311	Glycyrol	366.39	90.78	0.67	Gancao
12	MOL004904	licopyranocoumarin	384.41	80.36	0.65	Gancao
13	MOL005017	Phaseol	336.36	78.77	0.58	Gancao
14	MOL005970	Eucalyptol	266.36	60.62	0.32	Houpo
15	MOL013288	Picralinal	366.45	58.01	0.75	Huzhang
16	MOL000173	Wogonin	284.28	30.68	0.23	Huangqin
17	MOL001689	Acacetin	284.28	34.97	0.24	Huangqin
18	MOL007165	10α-cucurbita-5,24-diene-3β-ol	426.8	44.02	0.74	Gualou
19	MOL001924	Paeoniflorin	480.51	53.87	0.79	Zhimu

### Candidate Targets Screening and PPI Network Analysis

**Figure 5:**
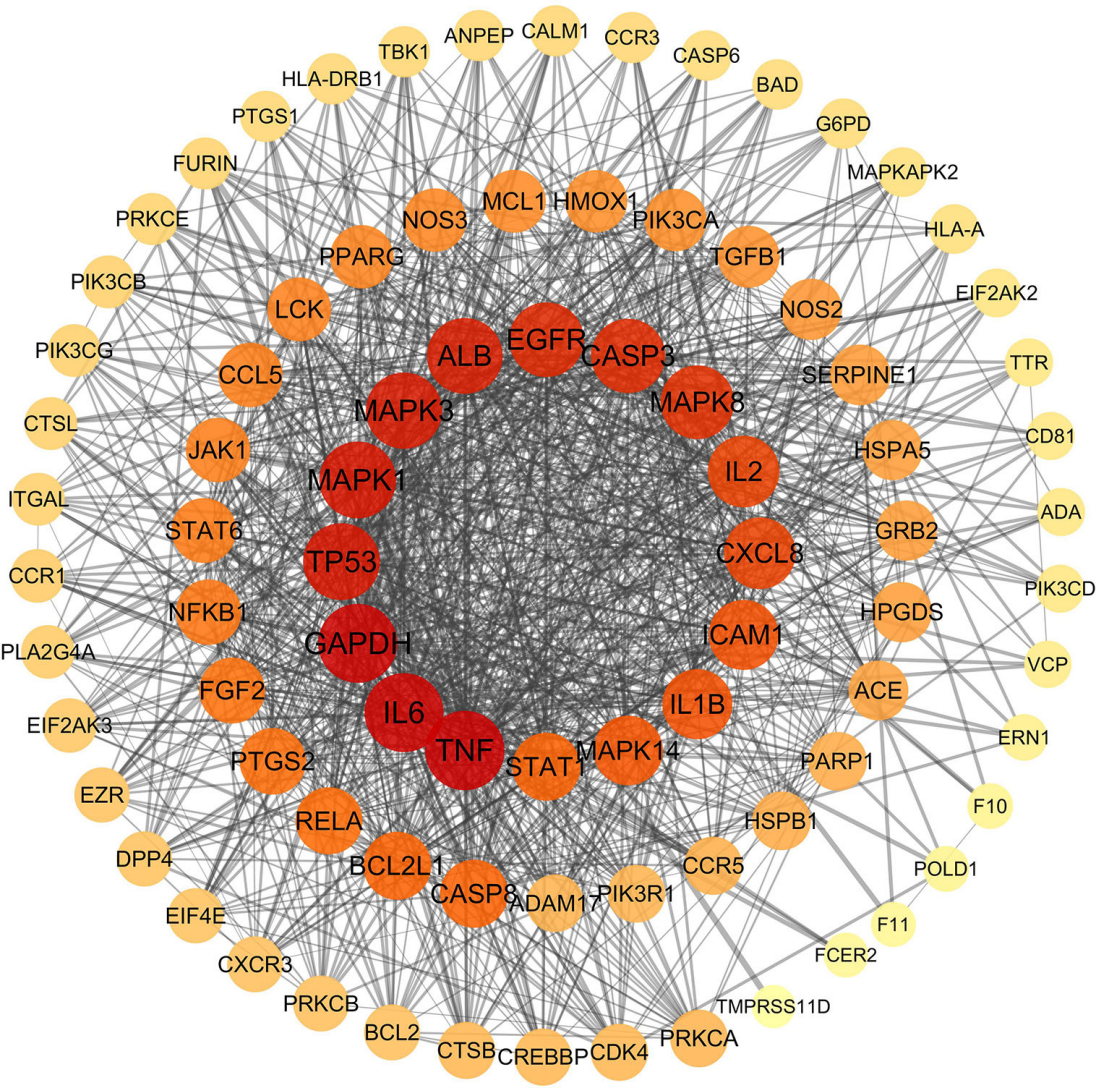
PPI network of candidate target. The colors of the nodes are illustrated from red to orange to yellow in descending order of degree values.

The SIB and TargetNet databases were used to screen 1116 targets corresponding to the 122 active components identified within QFDYG. A total of 259 targets relating to COVID-19 were obtained from GeneCards. In total, 85 common targets were identified as potential therapeutic targets for QFDYG against COVID-19. These targets were then uploaded into the STRING database to obtain information about the PPI, and then finally imported into Cytoscape for network analysis. Targets in the PPI network (**Figure 5**) with a high degree represented those with an important role in the central correlation. For example, the top 16 targets, ranked in order of degree, namely TNF, IL6, GAPDH, TP53, MAPK1, MAPK3, ALB, EGFR, CASP3, MAPK8, CXCL8, L2, ICAM1, IL1B, MAPK14, STAT1 tended to have more critical roles in the target network of QFDYG.

### GO Enrichment Analysis

**Figure 6:**
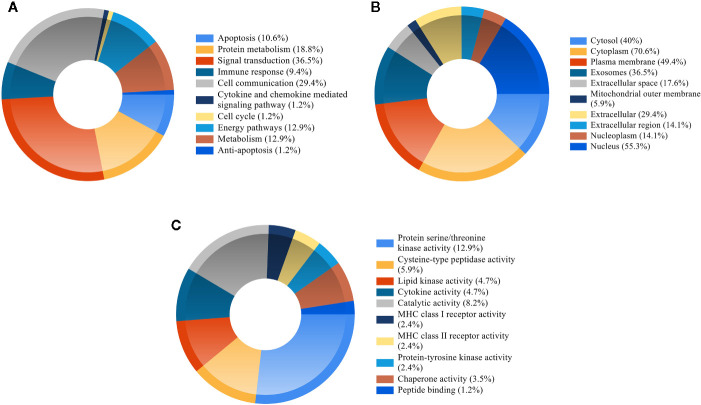
GO enrichment of the candidate targets. **(A)** Biological process of the candidate targets. **(B)** Cellular component of the candidate targets. **(C)** Molecular function of the candidate targets.

GO enrichment analysis of the 85 potential therapeutic targets was performed in order to identify relevant biological functions of QFDYG. The 10 most significantly enriched terms of a high percentage of genes in the BP, CC, and MF categories are displayed in **Figure 6**. BP analysis indicated that the targets were principally related to signaling transduction, cell communication, protein metabolism, energy pathway, metabolism, apoptosis and immune response. Analysis of the CC category indicated that the targets were mostly within the cytoplasm, nucleus, plasma membrane, cytosol, lysosomes and also extracellular. Analysis of MF indicated that the targets were mainly involved in activities related to serine/threonine kinases, catalysts, transcription factors, cysteine-type peptidases, lipid kinases and cytokines.

### KEGG Pathway Enrichment Analysis

**Figure 7:**
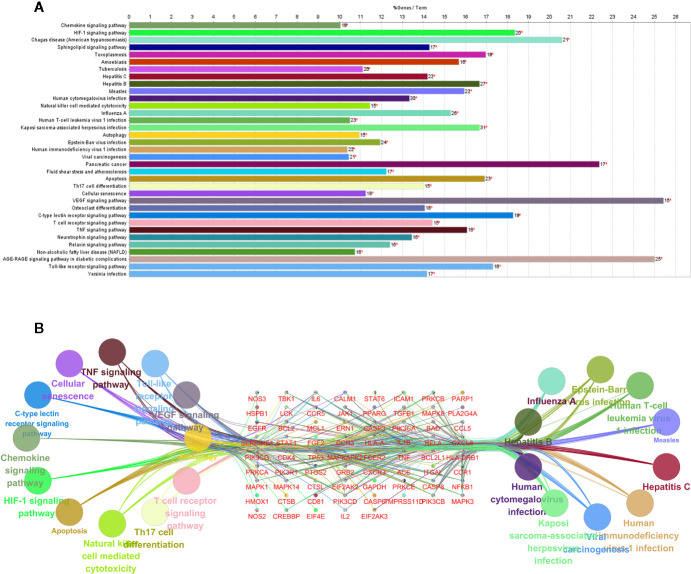
KEGG pathway enrichment of the potential targets **(A)**. Pathways and associated genes relationships network **(B)**.

To reveal the potential therapeutic mechanism of QFDYG on COVID-19, KEGG pathway enrichment analysis was conducted on 85 potential targets with 35 signaling pathways screened for those with *P-*values < 0.01, a kappa score ≥ 0.8, and numbers of genes ≥ 15. An overview map of the 35 KEGG signaling pathways is displayed in **Figure 7A**. KEGG pathway enrichment suggested that the targets were highly enriched in signaling pathways regulating virus infection and immune response, such as influenza A, hepatitis B, hepatitis C, Kaposi sarcoma-associated herpesvirus infection, human cytomegalovirus infection, viral carcinogenesis, human immunodeficiency virus 1 infection, Epstein-Barr virus infection, human T-cell leukemia virus 1 infection, the TNF signaling pathway, T cell receptor signaling pathway and Natural killer cell-mediated signaling pathway. The 11 most enriched signaling pathways were then selected to identify the relationship with their associated genes. As shown in **Figure 7B**, TNF, NFκB, IL-6, IL-1β, TP53, MAPK1, MAPK3 and MAPK14 were highly enriched.

### Drug-Compound-Target Network Construction

**Figure 8:**
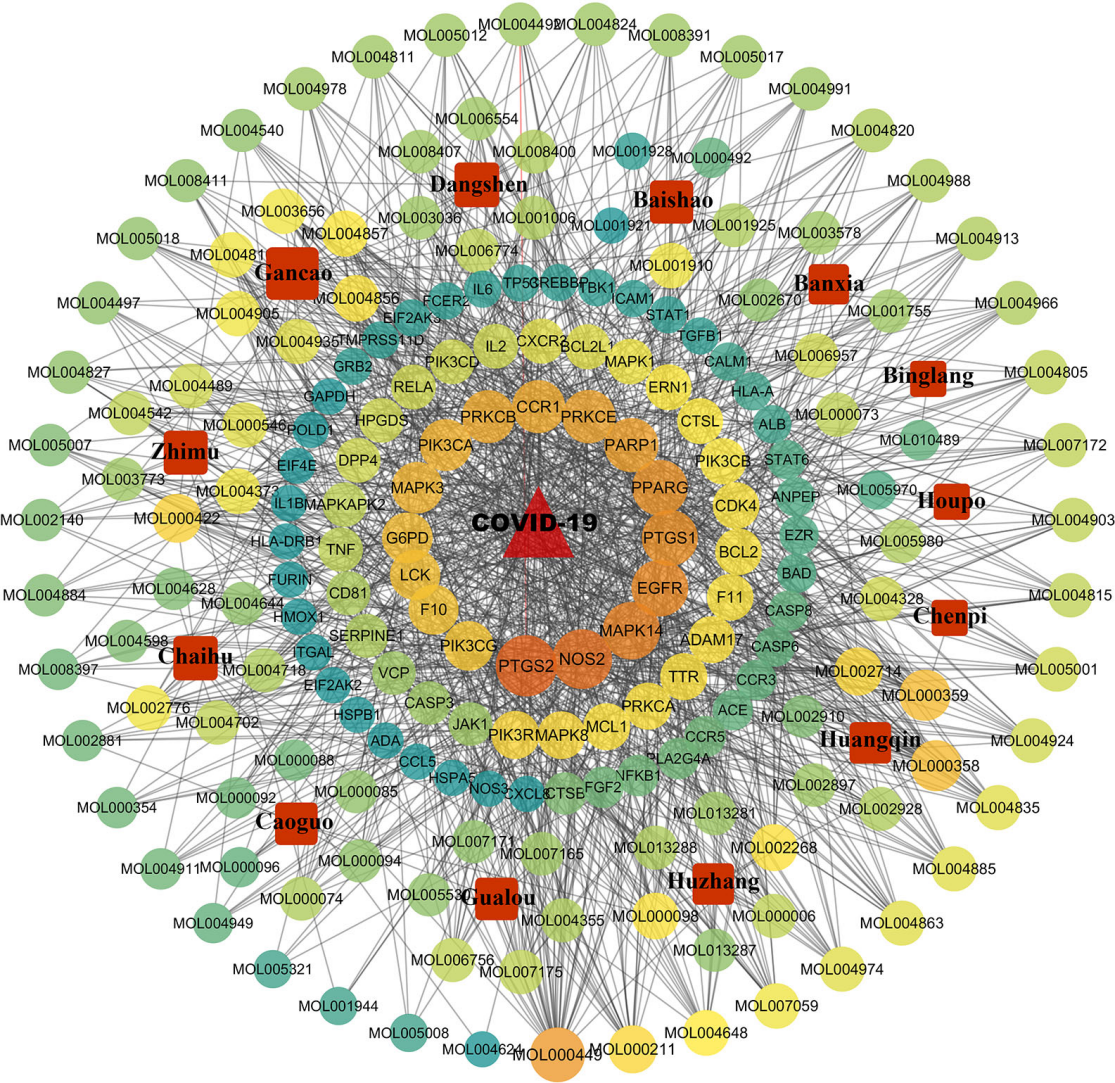
Drug-compound-target network of QFDYG. The red square nodes represent Chinese herbs. and the circular nodes represent active compounds and targets. Nodes size and color are illustrated from dark orange to yellow to green in descending order of degree values.

The 85 potential therapeutic targets and components and Chinese herbs within QFDYG were imported into Cytoscape 3.6.0 to construct a D-C-T network. The network (**Figure 8**) indicates that one Chinese herb contained multiple compounds, one compound acted on multiple targets, and one target was the target of multiple compounds, suggesting that the therapeutic characteristics of QFDYG were that of multiple components and multiple targets.

## Discussion

With the outbreak of COVID-19, there is an urgent need for specific and clinical antiviral regimens (Wu and McGoogan, 2020). However, there are no effective antiviral drugs for COVID-19, and the main strategy involves individualized treatment for symptomatic patients (Lai et al., 2020). Fortunately, in China, the combination of TCM treatment and effective life support was widely used in the early stage of the outbreak. The available data showed that the combination of TCM treatment with symptomatic treatment could reduce the transition of COVID-19 patients from mild to severe and promote the recovery from COVID-19 (Wan et al., 2020). QFDYG is a recommended TCM formula, which has been used clinically since the COVID-19 epidemic in Hubei Province. It has been reported that its clinical effect is better. We found that COVID-19 patients have the most common symptoms of fever and cough. It has been reported that fever is more than 80% and cough is more than 60%. Therefore, improving the symptoms of fever and cough is of great significance for the treatment of COVID-19 (Guan et al., 2020).

To clarify the clinical efficacy of QFDYG in treating COVID-19, a retrospective study was conducted, in which 103 patients were selected. Following the exclusion of 19 patients due to the clinical requirements of the study, data from 84 patients were analyzed in detail. Compared with the control group, combination treatment with QFDYG resulted in a significantly higher rate of recovery from symptoms over a shorter time. Nasopharyngeal swab tests for SARS-CoV-2 nucleic acid by RT-PCR is the principal text of effectiveness of COVID-19 treatment. The shorter the duration for a test to be negative, the better the drug treatment (Tahamtan and Ardebili, 2020). After 14 days of treatment, the proportion of negative nucleic acid tests in the combined treatment group was higher than that in the control group and the time was lower than the control group. QFDYG combined treatment, therefore, enhanced the negative rate and short recovery time of SARS-CoV-2 nucleic acid tests and shortened the duration of recovery. Chest CT is one of the vital evidences in the diagnosis for patients suspected of having COVID-19 infection, which improves the diagnosis rate and is also used to observe the patient’s treatment together with RT-PCR (Bernheim et al., 2020). Combination therapy with QFDYG shows better absorption of pulmonary inflammatory infiltration. Due to the complexity of COVID-19, other laboratory parameters such as hs-CRP, leukocyte count, neutrophil count and lymphocyte count are meaningful for clinical diagnosis as well (Soraya and Ulhaq, 2020). As a result, hs-CRP decreased significantly through the combination therapy with QFDYG compared with the data before admission, and what is more, the decrease rate was greater compared with the control group. Moreover, the lymphocyte count increased remarkably, indicating the obvious effect of the combination therapy with QFDYG on the recovery of the disease. No serious adverse events were reported in the clinical records, and there was no significant difference in the number of adverse reactions between the two groups, indicating that QFDYG treatment of COVID-19 was safe.

To further explore the foundation for the efficacy of QFDYG, network pharmacological approaches were integrated to investigate the bioactive components of QFDYG and their latent mechanisms for treating COVID-19. A total of 1643 components were obtained. Since antiviral components are absorbed *via* oral administration, the screening process for bioactive ingredients should include an assessment of drug-likeness (DL) and oral bioavailability (OB) (Yang et al., 2020b). The OB and DL of all compounds within QFDYG were extracted from the TCMSP database, finding that 122 compounds met the specified requirements. Targets are the key locations of disease treatment. Drugs can act both directly or indirectly on targets to fight a disease (Vinayagam et al., 2016). Therefore, all identified targets of compounds were compared with COVID-19 disease targets to obtain common cross-targets, of which 85 were finally identified. These common targets are considered potential therapeutic targets for drugs to treat COVID-19 disease and were used for further network pharmacological analysis.

Network pharmacological analysis indicated that the potential therapeutic targets of QFDYG were widely involved in mediating biological processes, such as inflammation, the immune response, both related to the body’s immune response after a viral infection. PPI network analysis indicated that TNF, IL-6, GAPDH, TP53, MAPK1, MAPK3, ALB, EGFR, CASP3, MAPK8, CXCL8, L2, ICAM1, IL1B, MAPK14 and STAT1 were located in the center of the network, and may represent hub targets of QFDYG. GO enrichment analysis suggested that the potential therapeutic targets in QFDYG may exert their therapeutic effect by regulation of signaling transduction, apoptosis, the immune response, and other biological processes *via* modulation of transcription factors and functional protease activity in the plasma membrane, cytosol, and nucleus. KEGG pathway enrichment analysis indicated that the 85 therapeutic targets were highly enriched in viral infection signaling pathways, such as influenza A and HIV-1 virus infection. They were also involved in immune respone, such as human T-cell, and activcation of the TNF signaling pathway, Tcell receptor signaling pathway and Natural killer cell-mediated signaling pathway. In the signaling pathway network, TNF, NF-κB, IL-6, IL-1β, TP53, MAPK1, MAPK3 and MAPK14 were the most enriched targets.

From the enrichment results of the KEGG pathway, 35 pathways were obtained, of which 10 were related to anti-viral action and 14 were related to the immune response. It can be inferred that the potential mechanism of action of QFDYG is mostly through anti-viral mechanisms and an immune response. SARS-CoV-2 is a type of β-coronavirus, a single-stranded positive-sense RNA virus that causes respiratory and enteric disease in humans and other animals (Wei et al., 2020). According to the results of this research, 6 Chinese medicines in QFDYG have antiviral effects, namely Chaihu, Huangqin, Houpo, Chishao, Gancao and Huzhang (Iore et al., 2007; Zhang et al., 2007; Shang et al., 2010; Xue et al., 2016; Yang et al., 2017). Components isolated from the Chinese medicine Chaihu, such as saikosaponins, have been reported to inhibit coronavirus and influenza virus activity, *via* an inhibitory effect of viral attachment, penetration and replication (Li et al., 2018). Baicalein, isolated from the Chinese medicine Huangqin can inhibit H1N1 virus activity by interfering with mRNA synthesis during the middle to late stages (Su et al., 2012). Similarly, glycyrrhizic acid isolated from the Chinese medicine Gancao can inhibit HIV virus activity. The mechanism of action may be to reduce membrane fluidity, reduce HIV-1-induced cell fusion and protein kinase C activity (Sun et al., 2019). Recent studies have demonstrated that SARS-CoV-2 displays a genomic organization similar to that of other β-coronaviruses, including the genes for three glycoproteins on the surface of the membrane, a spike protein (S), an envelope protein (E) and a membrane protein (M) (Li H. et al., 2020). The coronavirus enters cells by binding surface proteins *via* a receptor, causing infection and simultaneously inducing an immune response (Columbus et al., 2020). For example, MERS-CoV uses its S protein to bind to a specific dipeptidyl peptidase-4 receptor, which is considered a key factor in signal transduction and causes the activation of both the innate and specific immune response (Al-Tawfiq, 2020). The spike (S) glycoproteins of SARS-CoV-2 are no exception and mediate binding to host cells followed by membrane fusion (Chen W. et al., 2020). In the present study, molecular docking was used to establish that saikosaponins bind strongly to the S protein of the new coronavirus, saikosaponins U and V having the strongest binding, which is the subject of a potential future research study. From binding energy and interaction studies, Saikosaponins U and V displayed the strongest affinity towards the S protein (Sinha et al., 2020a). Similarly, a molecular docking simulation study of 20 compounds and an additional simulation study using molecular dynamics (MD) found that components of glycyrrhizic acid and glyasperin A displayed high binding affinity towards the S protein (Sinha et al., 2020b). COVID-19 principally spreads through the respiratory tract, and so the pharynx is a location that the virus can be found in high concentration. Samples were collected using nasopharyngeal swabs for an RT-PCR SARS-CoV-2 nucleic acid assay to determine whether the patients were infected with the virus, it is simple and reliable (Yang and Yan, 2020). The results of the present study indicate that combined treatment with QFDYG increased the proportion of positive to negative nucleic acid tests and reduced the time required for the nucleic acid test to become negative, indicating that QFDYG combination therapy may have a direct or indirect antiviral effect. From the results of these studies, we hypothesize that when treating patients positive for COVID-19, the active components of QFDYG, such as saikosaponins and glycyrrhizic acid, may enter cells infected with SARS-CoV-2, bind to membrane proteins, such as the S protein, regulate various pathways resulting in an anti-viral effect.

To date, seven types of human coronavirus (HCoV) have been found (Hasoksuz et al., 2020). Among them, Hcov-HKU1, HCoV-OC43, HCoV-NL63, and HCoV-229E mainly just cause common colds in adults and upper respiratory tract infections in children (Kong et al., 2020). The last three are SARS-CoV, MERS-CoV, and SARS-CoV-2. Although the pathogenesis of SARS-CoV-2 is not clear, the clinical manifestations similar to SARS-CoV and MERS-CoV suggest that the immune response plays an important role in the pathogenic mechanism (Pearson et al., 2019). The cytokine storm is an immune system in producing an uncontrolled and generalized inflammatory response (Mehta et al., 2020). SARS-CoV and SARS-CoV-2 infection can cause cytokine storm, accelerate the progression of systemic diseases, and cause multiple organ failure (Coperchini et al., 2020). The cytokine storm induced by abnormal immune activation was shown to be related to ARDS and respiratory failure in patients with COVID-19 (Conti et al., 2020). As showed in the KEGG pathway, chemokines, interleukins and TNF are associated with cytokine storm. Chemokines are a large family of cytokines characterized by a powerful chemotactic effect. Chemokines act as powerful chemoattractants (Rotondi et al., 2007). According to the gradient of chemokines, chemokines absorb inflammatory cells and make them migrate from the intravascular space through the endothelium and epithelium to the inflammation site (Zemans et al., 2009). Recently, several studies have investigated the role of chemokines in coronavirus associated infectious diseases. The results suggest that specific chemokines (such as CXCL8 and CXCL10) may play an important role in the development of COVID-19 related symptoms (Coperchini et al., 2020). Interleukins are related to the cytokine family of immune cell differentiation and activation (Scheller and Rose-John, 2006). They are widely involved in the expression and regulation of immune response, mediate the transport of immune cells to the site of infection, induce signal transmission in the acute phase, activate epithelial cells to induce the production of secondary cytokines, and play a key role in immune response (Hunter and Jones, 2015). Previous studies showed that the serum IL-6 level in patients with COVID-19 is increased, and its circulating level is positively correlated with the severity of the disease (Liu B. et al., 2020). Interleukin-6 (IL-6) is involved in the cytokine storm caused by coronavirus. It can be produced by B cells and T cells, and is closely related to the acute inflammatory phase. IL-1 β and tumor necrosis factor (TNF-α) can increase the production of this cytokine (Hunter and Jones, 2015). IL-6 may also be responsible for activating T helper cell 17 (Th17) in the interaction between dendritic cells and T cells. In patients with covid-19 infection, the high activation of Th17 cells may be caused by the increase of IL-6 production driven by the virus caused by the immune system (Kimura and Kishimoto, 2010). Due to its pleiotropy, IL-6 plays a key role in the pathogenesis of cytokine storm. TNF is mainly secreted by activated macrophages, NK cells, and T lymphocytes. TNF-α is a well-known typical proinflammatory cytokine and a key effector of a lethal cytokine storm. IL-1β and IL-6 are also proinflammatory cytokines. Diffuse alveolar injury is the main cause of respiratory dysfunction caused by a viral infection (Pirozyan et al., 2020). Continued disruption of the alveolar endothelial and epithelial barriers will finally lead to pulmonary alveolar-capillary barrier dysfunction. IL-1β and TNF can break the alveolar endothelial and epithelial barriers.

The innate immune system is deemed essential to preventing or inhibiting initial viral infections (Jawhara, 2020). The innate immune cells with antiviral activities are mainly phagocytes, dendritic cells, and natural killer cells. Innate immune cells usually use pattern recognition receptors (PRRs) to recognize pathogen-associated molecular patterns (PAMP) (Almeida et al., 2020). After the virus enters the infected cells through interaction with the receptor, it can further promote the innate immune cells to secrete cytokines and chemokines, such as IL-1β, IL-6, TNF-α, and IFN-β, which lead to the activation of inflammasomes (Yusuf et al., 2019). The phagocytes can phagocytize and degrade the viral particles. NK cells can secret granzymes and perforins, which induce autophagy and apoptosis of the infected cells (Zambello et al., 2020). Autopsies of COVID-19 patients showed apoptosis in the lung, spleen, and thyroid tissues (Hanley et al., 2020). In addition, SARS-CoV-2 infection can activate NF-κB, leading to multiple inflammatory and autoimmune diseases, which induces pro-inflammatory cytokines and chemokines, including IL-6, and recruits lymphoid cells and myeloid cells, such as activated T cells and macrophages (Hirano and Murakami, 2020). Studies have shown that nearly 20% of the active components in QFDYG have a therapeutic effect on pneumonia model, such as Baicalin, Emodin, Acacetin, Luteolin and Taraxerol et al. They can regulate the levels of IL-1β, IL-6, TNF-α inflammatory cytokines and chemokines, most of them can inhibit the expression of NF-κB signaling pathway to play an anti-inflammatory effect (Su et al., 2012; Dai et al., 2017; Khanra et al., 2017; Kong et al., 2019; Li S. et al., 2020). CT images show that most patients with COVID-19 have different degrees of infiltration in the lungs. The combined treatment of QFDYG can speed up the absorption of inflammatory infiltration in both lungs and promote the recovery of lungs to normal. It indicates that QFDYG may play a role in the treatment of pneumonia and reduced the mean time to symptoms recovery (cough and fever), by regulating the expression of inflammatory cytokines and the NF-κB signal pathway. IL-6 and TNF-α are inflammatory cytokines and the main inducers of C-reactive protein (CRP) secretion in liver (Liu F. et al., 2020). CRP is a sensitive biomarker associated with inflammation, infection, and tissue damage. In normal organisms, the protein expression level of CRP is low. During acute inflammation such as viral infections, the protein level rises rapidly, which is a non-specific acute-phase protein (Wang, 2020). Clinical results showed that combined treatment QFDYG with can effectively reduce the level of hs-CRP, which may reduce IL-6, TNF-α and other cytokines to play an anti-inflammatory or reduce cytokine storm effect. we speculate that QFDYG could treat COVID-19 by regulating the immune response and reducing the cytokine storm.

## Conclusion

In summary, QFDYG combination therapy confers a therapeutic effect on COVID-19 by both increasing the proportion of patients that recovered and accelerating the recovery from symptoms. QFDYG combined treatment increased the proportion of patients with a SARS-CoV-2 negative nucleic acid RT-PCR test and promoted focal absorption and inflammation on CT images. The mechanism by which QFDYG treats COVID-19 may be related to anti-viral action and regulation of an immune response and reduction of a cytokine storm. As an adjuvant therapy, QFDYG was beneficial for the treatment of COVID-19. However, due to the small sample size of this retrospective study, further double-blind, prospective, multi-center and randomized controlled trials are required to fully evaluate the clinical efficacy of QFDYG in a larger patient population. Additional investigation and experimental research are required to reveal the specific mechanisms and confirm its clinical efficacy.

## Data Availability Statement

The data used to support the findings of this study will be available from the corresponding authors upon request.

## Ethics Statement

The studies involving human participants were reviewed and approved by Institutional Ethics Board of Hubei Province Hospital of TCM. Written informed consent from the participants’ legal guardian/next of kin was not required to participate in this study in accordance with the national legislation and the institutional requirements.

## Author Contributions

YB, HW, YY, ZA and SZ: Clinical research program design and management. ZA, SZ and MW: Visualization and Software. WL, LW, GH, RT, XW, YS, LX: Clinical research plan execution and clinical data collection. ZA, WL and LW: Key revisions to the important knowledge content of the manuscript. All authors contributed to the article and approved the submitted version.

## Funding

This study was supported by Hubei Provincial Department of Science and Technology about New Pneumonia Emergency Project (No. 2020FCA007), Novel Coronavirus Pneumonia Emergency Science and Technology Project of Hubei University of Chinese Medicine (2020[NO.6]), and Academic Experience Inheritance of the Sixth National Group of Old Chinese Medicine Experts of the State Administration of Traditional Chinese Medicine (No. 2017 [29]).

## Conflict of Interest

The authors declare that the research was conducted in the absence of any commercial or financial relationships that could be construed as a potential conflict of interest.
